# The effect of exercise on ligament laxity during inversion/eversion rotations at the ankle joint

**DOI:** 10.1186/1757-1146-7-S1-A5

**Published:** 2014-04-08

**Authors:** Alison S Attenborough, Peter J Sinclair, Richard M Smith, Claire E Hiller

**Affiliations:** 1 Discipline of Exercise and Sport Science, The University of Sydney, Lidcombe, NSW, 2141, Australia; 2 Discipline of Physiotherapy, The University of Sydney, Lidcombe, NSW, 2141, Australia

## Background

Previous literature investigating the effect of exercise on ligament laxity at the knee found that basketball and distance running elicit a significant increase in knee laxity post exercise [1], whereas powerlifting [1] and cycling [2] do not change after exercise. This suggests that, to have an effect on ligament laxity, an activity must be weight bearing and repetitive in nature. We aimed to use a multidirectional exercise protocol to determine whether the ligaments responsible for controlling inversion/eversion at the ankle allowed greater rotation following dynamic movement. This will form the basis of future methodological decisions regarding the conditions under which laxity should be measured, and will help to describe the acute response of ligaments during exercise with applications to injury prevention.

## Methods

17 female volunteers (22.8 ± 2.3years, 165.4 ± 5.4m, 61.7 ± 8.3kgs) were tested on two separate mornings, having limited incidental activity and refrained from exercise. The order of the exercise and control session was randomised amongst participants, as was the leg tested. Ligament laxity was quantified as the joint rotation resulting from a 3Nmm torque applied in both an inversion and eversion direction using a Hollis Ankle Arthrometer (BlueBay Research, Milton FL). The exercise session involved 20min of physical activity separated into two identical 10min blocks that involved side stepping, agility tasks and jogging. Ankle laxity in the inversion/eversion plane was measured at baseline, following 10min of exercise and again following the second bout of 10min of exercise. The control session was identical to the exercise session however the exercise component was omitted in exchange for quiet sitting. A repeated measures ANOVA was used for analysis.

## Results

The magnitude of inversion/eversion rotation that resulted from the applied torques is presented in Table 1. There was a main effect of session (p=0.03) however no effect for time (p=0.07) or the interaction between session and time (p=0.14).

**Table 1:** Mean ± SD inversion/eversion rotation (degrees) at baseline, 10min into each session and 20min into each session.

	Baseline	10min	20min
Control	34.7 ± 8.1	34.3 ± 8.4	34.7 ± 11.4
Exercise*	35.8 ± 10.0	38.4 ± 9.5	39.9 ± 9.0

* p<0.05

## Conclusions

Multidirectional aerobic exercise increased the rotational movement at the ankle and implies that, with exercise, there is an acute mechanical response of the ligaments that support the ankle. The increase in laxity during exercise may explain the ankle sprain susceptibility during participation in sporting activities. To determine an individual’s baseline mechanical laxity, and ensure continuity between investigations, it is suggested that future measures are taken prior to engagement in physical activity.
